# A Possible Role for HMG-CoA Reductase Inhibitors and Its Association with *HMGCR* Genetic Variation in Parkinson’s Disease

**DOI:** 10.3390/ijms222212198

**Published:** 2021-11-11

**Authors:** Anna Pierzchlińska, Marek Droździk, Monika Białecka

**Affiliations:** 1 Department of Pharmacokinetics and Therapeutic Drug Monitoring, Pomeranian Medical University, 70-111 Szczecin, Poland; anna.pierzchlinska@pum.edu.pl (A.P.); monika-bialecka@post.pl (M.B.); 2 Department of Experimental and Clinical Pharmacology, Pomeranian Medical University, 70-111 Szczecin, Poland

**Keywords:** Parkinson’s disease, statins, HMG-CoA reductase inhibitors, genetic polymorphisms, neuroprotection

## Abstract

Parkinson’s disease (PD) is the second most common neurodegenerative disease characterised by both motor- and non-motor symptoms, including cognitive impairment. The aetiopathogenesis of PD, as well as its protective and susceptibility factors, are still elusive. Neuroprotective effects of 3-hydroxy-3-methyl-glutaryl-coenzyme A (HMG-CoA) reductase inhibitors—statins—via both cholesterol-dependent and independent mechanisms have been shown in animal and cell culture models. However, the available data provide conflicting results on the role of statin treatment in PD patients. Moreover, cholesterol is a vital component for brain functions and may be considered as protective against PD. We present possible statin effects on PD under the hypothesis that they may depend on the HMG-CoA reductase gene (*HMGCR*) variability, such as haplotype 7, which was shown to affect cholesterol synthesis and statin treatment outcome, diminishing possible neuroprotection associated with HMG-CoA reductase inhibitors administration. Statins are among the most prescribed groups of drugs. Thus, it seems important to review the available data in the context of their possible neuroprotective effects in PD, and the HMG-CoA reductase gene’s genetic variability.

## 1. Introduction

Parkinson’s disease (PD) is the second most common neurodegenerative disease characterised mainly by its motor symptoms, i.e., bradykinesia, tremor, rigidity, and postural instability. Along with motor impairment [1,2], PD patients may also suffer from non-motor symptoms, such as sleep disturbances, dysautonomia, and neuropsychiatric disorders, including depression and visual hallucinations [3]. In a substantial number of PD patients, cognitive impairment occurs, most often affecting visuospatial and executive functions [3,4]. Dementia, which may parallel the disease progression and increased neurodegeneration, is estimated to be six times more prevalent in PD than in age-matched general population, and is related to a loss of response to dopaminergic medications [3]. It substantially affects the quality of patient’s life, entails health economics and caregiver burden, and has a detrimental impact on life expectancy [5].

The aetiology of this devastating disease remains ambiguous despite a considerable number of preclinical and clinical studies. Available data indicate that age, gender, pesticides, and traumatic brain injury are seen as possible risk factors [6].

A misfolded protein—α-synuclein (α-syn)—constitutes the major component of intracellular and intraneuritic deposits typical for PD [7]. Although α-syn aggregation does not typically start in the substantia nigra, the PD motor symptoms become visible once dopamine neurons in this domain are affected (Stage 3 in the Braak system) [8]. A part of inherited forms of PD is caused by mutations in genes coding for α-synuclein (*SNCA*), leucine-rich repeat kinase 2 (*LRRK2*), parkin (*PRKN*), and PTEN-induced putative kinase 1 (*PINK1*) [7]. However, most of PD cases do not have a clear established background. Some factors, such as oxidative stress, neuroinflammation, proapoptotic mechanisms, excitotoxicity, and mitochondrial dysfunction have been suggested [6,9]. A multifactorial aetiology of PD seems to be plausible with mutual involvement of genetic and environmental factors [7]. Another obstacle to understand the PD pathogenesis may rely on the existence of overlapping symptoms of Parkinson’s disease dementia (PDD) and Alzheimer’s disease (AD). Thus, the clinical picture of some patients may resemble both AD and α-syn pathologies [10].

Evidence suggests neuroprotective effects of cholesterol-lowering agents—3-hydroxy-3-methyl-glutaryl-coenzyme A (HMG-CoA) reductase inhibitors (i.e., statins) [11]. However, clinical research conducted in PD patients assessing the impact of statin treatment on PD incidence did show conflicting results [12]. Moreover, there is a body of evidence determining high cholesterol levels as a protective factor in PD risks [13]. The relationship appears to be complex, since statin beneficial effects may depend on HMG-CoA reductase’s gene variability, haplotype 7, which results in reduced enzyme activity, diminished sensitivity to statins, and impaired cholesterol synthesis [14,15]. In the manuscript, we review a possible neuroprotective role of statins in Parkinson’s disease, considering the influence of haplotype 7 of the HMG-CoA reductase’s gene (*HMGCR*).

## 2. Pathogenesis of Dementia in Parkinson’s Disease

Cognitive decline is a frequent finding in PD patients with prevalence estimated at 20–30% for dementia (PDD) and over 60% for all executive functions. The cumulative prevalence of dementia is very high as at least 80% of patients with PD who survive >12 years from the diagnosis develop dementia [16]. Zhu et al. determined the following PDD risk factors: advanced age and fewer years of education, longer disease duration, later age-at-onset, higher levodopa (LD) use, more advanced Hoehn and Yahr disease stage, dyskinesia, excessive daytime sleepiness, autonomic dysfunction, depression, and hallucinations [17]. Available data demonstrate a multifactorial origin of PD dementia (pathological findings of Alzheimer-like and cortical Lewy body, metabolic and iatrogenic factors as dopaminergic or anticholinergic medications, blood pressure variability), and its identification may have an important prognostic value as well as potentially therapeutic role [18,19].

Parkinson’s and Alzheimer’s diseases are characterised by protein aggregation (with the majority of α-synuclein in PD and β-amyloid in AD) and inclusion body formation in the central nervous system (CNS) [20]. As it was stated above, clinical features of PD and AD may overlap—significantly more severe AD pathology (including cortical amyloid plaque load) is more often observed in PDD than in non-demented PD patients [21]—thus suggesting common pathogenic mechanisms of AD and PDD. Therefore, it seems justified to investigate the similar risk and protective factors of both disorders.

## 3. Protective Role of Cholesterol in Neurodegenerative Diseases

Approximately 25% of total body cholesterol resides in the brain, from which the major part constitutes myelin surrounding axons (70%) and plasma membranes of astrocytes and neurocytes [22,23]. Only a small amount of brain cholesterol is located within intercellular space and in cerebrospinal fluid (CNF), being associated with apolipoproteins, mainly apoE [22]. Nearly all cholesterol present in the CNS is produced in situ, since plasma lipoproteins do not cross the blood–brain barrier (BBB) [23]. Cholesterol is synthesised in the isoprenoid biosynthetic pathway, which starts from acetyl-CoA as a substrate and involves at least 20 enzymes [22]. Cholesterol plays an essential role in normal brain functioning, including memory and learning, neurotransmitter receptor expression, peripheral signalling, and antioxidant transport, such as coenzyme Q10 [15].

The protective role of cholesterol against PD was suggested by Huang et al. [13]. The authors found an association between lower levels of low-density lipoprotein cholesterol (LDL-C) and higher occurrence of PD. One of the recent prospective studies involving a cohort of over 250,000 individuals has shown that both higher total cholesterol and LDL-C levels decreased the PD risk. Nevertheless, the finding reached significance only in men [24]. A modest tendency of slower PD progression in subjects with higher total cholesterol levels has also emerged [25]. There are two possibilities of explaining those relationships: lower levels of cholesterol may result from malnutrition and smaller exogenous cholesterol intake [26] or from its reduced synthesis due to decreased activity of HMG-CoA reductase, which was defined in fibroblasts from patients with PD [27]. The correlation between serum and brain levels of cholesterol is still ambiguous and measurable only indirectly. In some analyses, hydroxylated form of cholesterol—24-hydroxycholesterol—was decreased in the plasma of PD patients. It is the main form of cholesterol excreted from the brain, and its level may reflect brain cholesterol metabolism. Furthermore, the phospholipids to cholesterol ratio in membrane microdomains, called lipid rafts, was increased in the frontal cortex of PD patients’ brains, indicating a net reduction of cholesterol content [28].

Since cholesterol is vital to normal brain functioning, the impact of its levels on cognitive functions has been examined. In one study, baseline higher serum LDL-C levels among PD patients were associated with better cognitive performance in statin non-users, but not in statin users. However, small sample size of this subgroup (18 participants) could affect the observations [26]. The protective impact of high cholesterol levels on PDD was established in the Sławek et al. research, although no information on statin treatment was provided [29]. 

Nevertheless, it needs to be considered that even if higher cholesterol levels are beneficial for cognitive functions, high LDL-C levels may increase risks of cardiovascular diseases [26]. Thus, administration of lipid-lowering medications and its possible consequences should be carefully analysed.

## 4. Detrimental Role of Cholesterol in Neurodegenerative Diseases

Nonetheless, there are uncertainties over the role of cholesterol in the development of neurodegenerative diseases. Cholesterol has been determined to enhance α-syn and β-amyloid aggregation, while metabolites of cholesterol (oxysterols) were associated with oxidative stress and inflammation in neuronal cells or even apoptosis of dopaminergic neurons [30].

The negative impact of high cholesterol levels was demonstrated in a prospective Finnish study [31]. Accordingly, Anstey et al., in their meta-analysis, correlated the risk for dementia and cognitive impairment with higher total cholesterol levels. However, the study population did not include PD cases [32]. High cholesterol levels are often accompanied by other vascular risk factors, such as obesity, glucose intolerance or hypertension, described together as metabolic syndrome. In one of the recent studies metabolic syndrome has been determined as a risk factor for mild cognitive decline (MCI) and dementia in PD, along with total cholesterol level as an individual factor alone [33]. Additionally, the authors also analysedtreatment of metabolic syndrome—treated patients exhibited lower dementia risk than the untreated ones. On the other hand, in several studies no associations between cognitive functions or dementia in PD and cholesterol levels were found [34,35].

## 5. Statins and HMG-CoA Reductase

Statins are commonly prescribed as lipid-lowering agents, serving as protective agents in cardiovascular diseases. A wide range of their beneficial effects, including cholesterol-independent mechanisms, has resulted in statins being one of the most prescribed groups of drugs [36]. The statin family consists of eight drugs: mevastatin and lovastatin, the first studied in humans; then pravastatin and simvastatin, derivatives of lovastatin; and atorvastatin, fluvastatin, pitavastatin, and rosuvastatin, synthetic compounds [37]. Statins comprise modified 3,5-dihydroxyglutaric acid moiety, which is structurally similar to an endogenous compound, i.e., 3-hydroxy-3-methyl-glutarylcoenzyme A (HMG-CoA) [38]. Statins act by competitive inhibition of HMG-CoA reductase (therefore their full name is HMG-CoA reductase inhibitors) in the above-mentioned metabolic pathway resulting in interference with HMG-CoA conversion into mevalonate, and thus decreased endogenous cholesterol levels [39]. Finally, it leads to upregulation of LDL-C receptor expression in hepatocytes, which is followed by LDL-C uptake and a net reduction of its plasma level [40]. Statins reduce triglycerides, and to a much lesser degree, increase high-density lipoprotein cholesterol (HDL-C) levels [37]. What is important, the inhibition of HMG-CoA reductase does not cause generation of potentially harmful cholesterol precursors since water-soluble hydroxymethylglutarate—HMG-CoA product of reversible reaction catalysed by transferase—can serve as a substrate in other metabolic pathways [41].

The pharmacokinetics of statins is determined by their lipophilicity, resulting from nonpolar substituents, and other structural properties—the presence of inactive lactone form converted into an active metabolite (simvastatin, lovastatin) or active acid form (atorvastatin, rosuvastatin, pitavastatin, fluvastatin and pravastatin) [38,42]. The metabolism of some statins involves cytochrome P450 (CYP450) system—a family of enzymes crucial for oxidative metabolism of several drug classes, as well as endogenous substances. The highest activity of CYP450 enzymes is defined in hepatocytes [43]. Atorvastatin, simvastatin and lovastatin are substrates of CYP3A4, a CYP450 isoenzyme that oxidises the majority of drugs, whereas fluvastatin is metabolised by CYP2C9. On the other hand, pravastatin, pitavastatin and rosuvastatin are not metabolised by any of the CYP450 enzymes [38,42].

Statins may cross the BBB depending on their lipophilicity, thus pravastatin and rosuvastatin have a minimal permeability [44]. However, fluvastatin does not cross the BBB either, in spite of its amphiphilic character [45]. On the other hand, the lipophilicity theory does not seem to be definitive, since lipophobic statins were found in the brain of animal models and in human cerebrospinal fluid (CSF) [36]. Thus, other mechanisms than passive diffusion have been suggested to transport lipophobic statins into the CNS, such as organic anion transporting polypeptides (OATP) or monocarboxylic acid transporters (MCT) [36,46].

## 6. Statins in Neuroprotection

A number of evidence on antioxidant, anti-inflammatory and anti-excitotoxic properties of statins has been shown [11], which may not only be based on cholesterol-dependent mechanisms. As an example, statins may improve vascular function and increase cerebral blood flow. LDL-C downregulates the production of endothelium-derived nitric oxide (eNO), a well-known vasodilator. Statins do not only reduce LDL-C level, but also activate protein kinase B (Akt/PKB), which in turn induces eNO production by Akt-mediated phosphorylation of eNO synthase (eNOS) [47,48].

Isoprenylation with isoprenoid intermediates: farnesylpyrophosphate (FPP) and geranylgeranyl pyrophosphate (GGPP) is vital for intracellular transport and function of small GTP-binding proteins. The protein, depending on its structure, can be either isoprenylated with FPP (Ras family proteins) or GGPP (Rab and Rap family proteins). However, Rho family proteins can be post-translationally modified with GGPP only, or with both intermediates. Statins block synthesis of the isoprenoid intermediates, thus prevent isoprenylation and inhibit function of signalling molecules [49]. It seems important, as Rho activation downregulates eNOS expression. Furthermore, statins increase half-life of eNOS mRNA and prevent its destabilisation, which can be caused by tumour necrosis factor-alpha (TNF-α), oxidised LDL-C, and hypoxia. The effect of statins was reversed by GGPP, but not by FPP or LDL-C, suggesting cholesterol- and FPP-independent mechanism [49,50].

Statins improved stroke outcome in wild-type animal models, but not in eNOS-deficient mice, indicating eNO-based mechanisms [47,51]. However, other types of nitric oxide, namely inducible nitric oxide (iNO) or neuronal nitric oxide (nNO), have detrimental consequences for surrounding tissues. Ischaemia induces iNO and nNO synthesis, which in turn leads to toxic concentrations of NO in neurons and further aggravates the ischaemic damage [47]. iNO was correlated with neuroinflammation in PD. Moreover, tyrosine nitration of α-syn accelerated its aggregation [52]. Statins downregulate the activities of both iNO and nNO synthases [53].

In addition to improvement of vascular functions, statins can reduce oxidative stress, which is presumably involved in the pathogenesis of several neurodegenerative diseases, such as AD and PD [54]. Oxidative stress highly correlates with overproduction of reactive oxygen species (ROS), synthesised mainly by NADPH-dependent oxidases [11,55]. ROS may be converted into a reactive hydroxyl ion (OH-) and peroxynitrite, which produce damage to cell constituents: lipids, proteins as well as nucleic acids, thus inducing ischaemic cell death [56]. Moreover, oxidative stress upregulates non-enzymatic production of oxidised cholesterol derivatives—oxysterols, i.e., 7-ketocholesterol (7-K), 7β-hydroxycholesterol (7β-OH), 5α,6α-epoxycholesterol (α-EPOX) and 5β,6β-epoxycholesterol (β-EPOX) [57]. In PD, 7-K and 7β-OH plasma levels (along with an enzymatic product of cholesterol oxidization—27-hydroxycholesterol) were significantly elevated compared to the control group, indicating an association between PD and oxidative stress [58]. Statins can downregulate ROS production by inhibiting NADPH-complex [53,55]. Furthermore, pitavastatin was shown to protect against ischeamia-induced neuronal damage by preservation of the antioxidant enzyme—superoxide dismutase (SOD) [59]. On the other hand, inhibition of HMG-CoA reductase may result in a decrease in isoprenoid ubiquinone or coenzyme Q10 levels that perform antioxidative functions [53].

Inflammation in the CNS involves chronic activation of glial cells—microglia and astrocytes. Microglia serve as the CNS immune response cells. Upon activation they produce proinflammatory cytokines, such as TNF-α or interleukin-1β (IL-1β), which were found to be elevated in the substantia nigra of PD patients [9]. Moreover, activated microglia were described as clustered around dystrophic dopamine neurons [60]. In an animal PD model, atorvastatin not only decreased TNF-α and IL-1β levels in the striatum but also prevented overproduction of ROS [61]. McFarland et al. discovered in in vitro study using lipopolysaccharide (LPS)-induced neuroinflammation, that pretreatment with atorvastatin, pravastatin, or rosuvastatin reduced TNF-α levels and prevented cell death. Interestingly, rosuvastatin did not lead to a decrease in IL-1β levels, which may imply IL-1β-independent neuroprotective mechanisms of the drug [62].

The release of several inflammatory cytokines, such as TNF-α, IL-6, and IL-1, is regulated by transcription nuclear factor-κB (NF-κB) [56], which is activated in the substantia nigra pars compacta (SNpc) of PD patients and PD animal models [63]. Ghosh et al. showed in an experimental study, using a well-established neurotoxin 1-methyl-4-phenyl-1,2,3,6-tetrahydropyridine (MPTP), that NF-κB required a small G-protein, p21ras for its activation. Simvastatin, by inhibiting p21ras, decreased activation of NF-κB, thus significantly lowering TNF-α, IL-1β and iNOS expression. The treatment diminished microglial and astroglial activation. Presumably, the effect on p21ras was related to inhibition of the protein isoprenylation. However, it should be stated that, according to some research, simvastatin in high doses may exert deleterious impact on the nigrostriatum [63].

A beneficial effect of statin treatment may also be produced by inhibition of α-synuclein aggregation. In the analysis carried out by Bar-On et al., statins, i.e., lovastatin, simvastatin, and pravastatin, diminished α-syn aggregation in the detergent-insoluble fraction of a neuroblastoma cell line. Similar results were obtained using lovastatin in mature human neurons transfected with lentiviral vectors expressing α-syn [64]. Several probable mechanisms, as the findings explanation, have been suggested—first of all, a decrease in cholesterol level may result in a depletion of lipid rafts, which α-syn is associated with [65]. Furthermore, oxidative stress is responsible for synthesis of oxysterols that enhance α-syn aggregation [11]. Thus, it is possible that statins ameliorate α-syn accumulation through their antioxidative properties. An example was demonstrated in an animal model—lovastatin decreased level of oxidised α-syn and reduced its accumulation [66]. Finally, oxidative stress may lead to post-transcriptional modification of α-syn via nitrative radicals, such as iNO, which was associated with α-syn aggregation [64,66].

**Figure 1:**
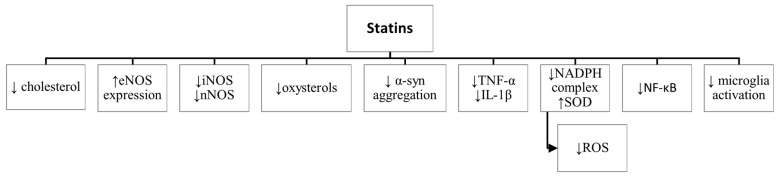
Neuroprotective mechanisms of statins. ↑—increase, ↓—decrease, eNOS—endothelial nitric oxide synthase, iNOS—inducible nitric oxide synthase, nNOS—neuronal nitric oxide synthase, α-syn—alpha-synuclein, TNF-α—tumor necrosis factor-alpha, IL-1β—interleukin-1beta, NADPH—nicotinamide adenine dinucleotide phosphate, SOD—superoxide dismutase, ROS—reactive oxygen species, NF-κB—transcription nuclear factor-kappaB.

Summarising, statins exerted neuroprotective effects both through cholesterol-dependent as well as cholesterol-independent mechanisms, including vasorelaxation, anti-inflammatory, and antioxidative functions (Figure 1).

## 7. Statins in PD

Despite many studies describing beneficial effects of statins in neuroprotection, there are conflicting results on the role of HMG-CoA reductase inhibitors in Parkinson’s disease. The latest meta-analysis included 17 observational studies: 8 case-control and 9 cohort studies [12]. The predominant number of the cited observations are in favour of statin use, demonstrating lower PD occurrence in statin users with a summary OR = 0.92 (95% CI: 0.86–0.99). However, different types of statins exerted diverse impact on PD incidence, with atorvastatin showing the most beneficial effect. The results of another meta-analysis suggested an association between pravastatin use and PD occurrence, presumably due to its lowest lipophilicity, thus difficulties to cross the BBB [39]. Statins in PD subjects increased performance in cognitive functions, i.e., global cognition, verbal fluency, and executive processing [67]. Considering the lipophilicity of HMG-CoA reductase inhibitors used in the analysis, better scores in the Montreal Cognitive Assessment (MoCA) test were observed in the case of lipophilic statin users than in lipophobic ones. Moreover, longitudinally, patients medicated with lipophilic statins declined slower in Dementia Rating Scale-2 (DRS) than non-statin users. In a recent prospective study, statins also have shown protective effect against motor symptoms progression, with the highest impact on rigidity [68].

Numerous issues have arisen concerning the available information. First, several of studies did not analyse a vital confounding factor, i.e., LDL-C level. Huang et al. indicated that high LDL-C levels, established to be associated with lower PD occurrence, trigger statin prescription [25]. Thus, statin advantages may result from beneficial effects of higher cholesterol levels, and to a lesser extent to statin treatment itself. In addition, regarding high LDL-C levels as a PD protective factor, it is likely that the use of HMG-CoA reductase inhibitors, decreasing LDL-C, could be inversely associated with PD occurrence. Indeed, in the meta-analysis reported by Bykov et al., the protective effect of statins on PD was observed when no adjustment for cholesterol levels was applied, and disappeared after the adjustment [69]. Other confounding factors that could influence findings, and were omitted in several studies, included comorbidity of diabetes mellitus or medications, such as nonsteroidal anti-inflammatory drugs (NSAIDs), anti-diabetics, or calcium channel blockers [70]. The characteristics of PD, namely a rather slow progression with a long presymptomatic period, is a crucial obstacle in the PD study design. Moreover, an impact of variability in HMG-CoA gene (*HMGCR*) should be taken into consideration since it can alter statin treatment responses but may also be involved in PD susceptibility. So far, no study has analysed possible associations between *HMGCR* genotype, statin treatment, and PD or PDD risks altogether.

## 8. *HMGCR* Gene Polymorphisms and Haplotypes

Although statins’ lipid-lowering features have been proven, it is assessed that almost a third of patients do not reach treatment goals. Besides the possibility of insufficient dosage, there are several other factors that may affect treatment outcomes. The response to HMG-CoA reductase inhibitors depends on patient’s age, environmental factors, such as smoking status, body weight, physical activity, and diet, but also on genetic factors—ethnicity and polymorphisms in *HMGCR* [71]. In the study performed by Chasman et al., two common and closely linked *HMGCR* single nucleotide polymorphisms (SNPs) (named in the study SNP 12 and SNP 29) were associated with 22% smaller reduction in total cholesterol, and 19% smaller reduction in LDL-C plasma levels in patients treated with pravastatin for 24 weeks [72]. It was suggested that minor alleles of the intronic SNPs (rs17244841 and rs17238540, respectively) defining haplotype 7 (H7), correlated with altered response to pravastatin therapy. Haplotype 7 was later redefined by Krauss et al., by an additional intronic SNP 20144 (rs3846662), also present in haplotype 2 (H2) of *HMGCR* gene [40,71,73]. Among simvastatin users a significant impact of H2 and H7 on reduced LDL-C change was observed only in subjects of African or African American ancestry. The lowest response to the treatment was noticed in carriers of combined H2 + H7 haplotypes, who were almost entirely of non-Caucasian ancestry. Haplotype 2 is observed much more frequently among subjects of African ancestry than in Caucasians (32% vs. 2%); thus, the ethnic factor may be responsible for the observed differences [71].

Haplotype 7 prevalence is defined in 3% of Caucasians and 6% of African American, and though all H7 SNPs are located in the introns, a crucial impact on HMG-CoA activity is observed. H7 is strongly associated with an alternative splicing product lacking exon 13 (*HMGCR13*(−)). Since the substrate-binding domain of HMG-CoA reductase is encoded, among others, by exon 13, it is possible that *HMGCR13*(−) has a deleterious impact on statin affinity. In fact, mRNA expression analysis in simvastatin-incubated immobilised lymphocyte cell lines (derived from simvastatin-treated patients) revealed a correlation between lower reduction of plasma total cholesterol and LDL-C levels with higher expression of *HMGCR13*(−). Accordingly, the alternative splicing of *HMGCR* was associated with reduced mRNA upregulation of LDL-C receptor gene, and a weaker statin response [40]. However, it needs to be considered that HMG-CoA reductase forms a tetramer comprised of two dimers. Hypothetically, each of the monomers can vary between *HMGCR13*(+) and *HMGCR13*(−) form, thus the resulting enzyme activity may be only partially attenuated [74].

Up to date, most of studies analysed individual SNPs in *HMGCR* gene and their influence on statin responses. The major A allele of *HMGCR* rs3846662 polymorphism strongly correlates with the splicing defect and results in high prevalence of the *HMGCR13*(−) transcript. Thus, it has been suggested that A allele carriers may respond worse to the treatment. Indeed, in the Leduc et al.’s study in familial hypercholesterolaemia, the patients homozygous for the A allele experienced significantly lower reduction in LDL-C levels, but the relationship was observed only in women [75]. The baseline total cholesterol and LDL-C levels were higher in all patients carrying the AA genotype. However, since the population comprised subjects with heterozygous familial hypercholesterolaemia, statin treatment response could be affected by reduced activity of LDL-C receptors. The same variant of *HMGCR* gene was investigated in dyslipidaemic patients, yet the results showed an inverse relationship, as the G allele carriers were less likely to reach the treatment goal of total cholesterol reduction [76]. In a Korean study, the rs3846662 GG genotype carriers had significantly higher baseline LDL-C levels than AA homozygotes with a similar tendency during atorvastatin treatment [77]. Nevertheless, the significance level decreased after compressing the initial range of LDL-C levels between study groups.

Another polymorphism, which haplotype 7 includes—*HMGCR* rs17238540—was analysed for statin response in diabetic patients [78]. The treatment goal (total serum cholesterol level of 4 mmol/L or less) was achieved in 72% of homozygotes for the major T allele. However, only 49% of the minor G allele carriers reached the aim of the treatment. This group also experienced lower reduction in total cholesterol, as well as in triglycerides levels. No significant correlation between *HMGCR* rs17238540 and the baseline levels of total cholesterol, LDL-C, HDL-C or triglycerides levels was observed.

Considering conflicting results on the relationship between the polymorphisms of *HMGCR* gene and statin treatment response, the available data do not facilitate a simple understanding.

## 9. *HMGCR* Genetic Variability in PD and Other Neurodegenerative Diseases

Single nucleotide polymorphisms (SNPs) in the gene coding for HMG-CoA reductase have recently been investigated in neurodegenerative diseases, mostly in Alzheimer’s disease. As mentioned above, haplotype 7 may downregulate the protein activity, LDL-C synthesis and affect statin treatment outcome [14]. Therefore, provided that higher cholesterol levels serve as protective factors in PD, *HMGCR* lacking exon 13 may constitute a susceptibility factor. On the other hand, unresponsiveness to HMG-CoA reductase inhibitors of *HMGCR13*(−) carriers could diminish their neuroprotective role describedin the previous paragraphs. The majority of studies analysed rs3846662: A > G polymorphism and defined a protective impact of the major A allele against AD or conversion of MCI to AD [14,75,79,80]. Leduc et al. in their study in 324 autopsy-confirmed cases of AD showed that in women the AA genotype of rs3846662 was protective against sporadic AD (OR = 0.521; *p* = 0.0028), and delayed its onset by 3.6 years (*p* = 0.017). In another cohort of patients, the authors tested the risk of conversion from MCI to AD. While there was no impact of the rs3846662: AA alone, it significantly decreased the risk of the conversion in patients carrying *APOE4*. The *APOE4* allele codes a variant e4 of a lipid carrier protein apolipoprotein E, and was strongly associated with the risk of AD, MCI to AD conversion, and decreased age at AD onset [14]. Wright et al. performed a similar study in 490 AD subjects, which showed a protective effect of the AA genotype of rs3846662 (*p* = 0.049), even stronger in male and female *APOE4* carriers (*p* = 0.016). However, the authors did not find an association between rs3846662 and the risk of MCI to AD conversion, in either of *APOE4* allelic groups [80]. Keller et al. and Licastro et al. examined another functional polymorphism, −911C > A (rs3761740) [81,82], located in *HMGCR* gene promoter, possibly near a binding site for SREBP-2 (sterol regulatory element-binding protein 2), a membrane-bound transcription factor capable of increasing *HMGCR* transcription [81]. In fact, the polymorphism was associated with upregulated SREBP-2 sensitivity in in vitro experiment. Although Keller et al. reported no correlation between the risk of AD and rs3761740 alone among *APOE4* carriers, the minor A allele of rs3761740 increased the risk by 36% (OR = 6.21; *p* < 0.001) compared to the group carrying only *APOE4* (OR = 4.57; *p* < 0.001) [81]. Licastro et al. analysed several gene variants that favour inflammation, including *APOE4* and *HMGCR* rs3761740 in 260 AD subjects [82]. The authors identified four risk sets: with a low intrinsic risk of AD, with early onset AD (having more rapid cognitive decline), and two late-onset AD (divided according to age at onset: 65–74 and 75+ years). The early-onset group, as well as the group being affected at the age of 65–74, were more likely to be heterozygous for the *HMGCR* SNP. Moreover, in the latter group the AA genotype of *HMGCR* was more prevalent than in other risk groups. The *APOE4* allele was over-represented in both late-onset groups. Interestingly, *HMGCR* SNP was assessed to be more informative, displaying more different frequencies from group to group than *APOE4* genotype. Rodriguez-Rodriguez et al. studied the same *HMGCR* SNP along with *HMGCR* rs3931914: G > C located in the untranslated region (5′UTR) of the gene [83]. None of the SNPs analysed alone exhibited an impact on AD incidence, yet the risk was significantly greater among wild-type homozygotes of rs3931914 expressing either *ABCA1* (cholesterol transporter gene) polymorphisms: rs1800977 (OR = 2.77; *p* = 0.02) or rs2422493 (OR = 2.07; *p* = 0.02). Genetic variants of *ABCA1* were implicated in modifying the risk of AD and influencing CSF level of 24-hydroxycholesterol—a possible marker of brain cholesterol metabolism [28,83]. So far, only two studies have examined the impact of *HMGCR* variability on PD [84,85]. Benn et al. analysed rs17238484—a polymorphism downregulating cholesterol synthesis—in participants of two prospective Danish studies [84]. Among over 111,000 subjects, 1001 developed AD, 256 vascular dementia, 2154 any dementia, and 460 PD (no information on cognitive status of PD patients) during 37 years of follow-up. The authors did not find a correlation between the polymorphism and any of those disease states. An increased hazard ratio (HR) for PD in subjects with LDL-C level < 1.8 mmol/L vs. ≥ 4.0 mmol/L (HR = 1.70; *p* = 0.01), indicating a possible role of *HMGCR* activity and a neuroprotective impact of high cholesterol levels was observed. A recent study has investigated an impact of 6 *HMGCR* polymorphisms on PD and 21 other non-vascular diseases [85]. The authors calculated a weighted genetic risk score (GRS), which included rs17238484, but none of the polymorphisms within haplotype 6. The results suggested a correlation between GRS, linked to a decrease in LDL-C levels, and PD, however, without statistical significance after the Bonferroni correction. Nevertheless, the study showed that lower LDL-C levels should not be simply taken as beneficial in the case of PD.

## 10. Conclusions

**Figure 2:**
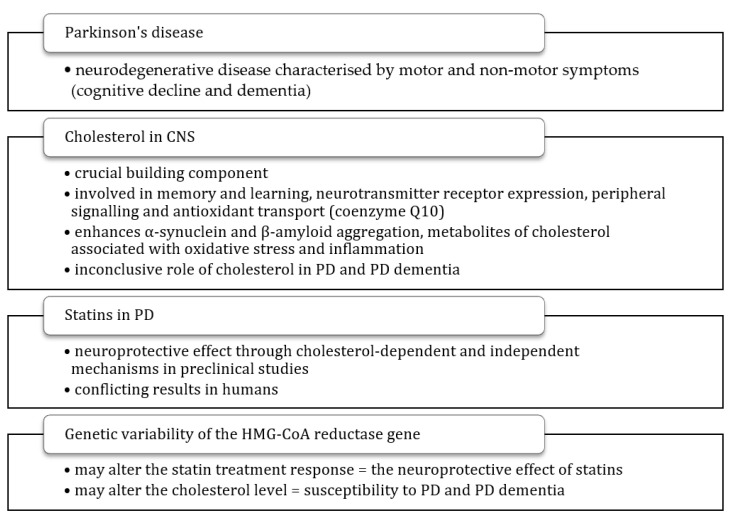
Summary of a potential role of cholesterol, statins, and genetic variability of the HMG-CoA reductase gene in Parkinson’s disease. CNS—central nervous system, PD—Parkinson’s disease.

In a society with a constantly increasing median age, it is necessary to understand the aetiology of neurodegenerative diseases such as PD, as this knowledge may help to develop an effective treatment approach. HMG-CoA reductase inhibitors are commonly used drugs, which were shown to provide neuroprotective effects in animal and cell culture models, yet statin treatment is not considered as protective against PD. In fact, several studies investigated the role of statin treatment in PD susceptibility with conflicting results, not reflecting the possibilities demonstrated in preclinical observations. Another obstacle constitutes the ambiguous role of cholesterol in the pathogenesis of PD. The available knowledge on the role of cholesterol and statins in PD, impact of *HMGCR* polymorphisms on statin treatment, and questions that have not been answered yet are summarised in Table 1 and Figure 2. Up to date, there has been no study assessing the impact of *HMGCR13*(−), which is suggested to affect statin treatment outcome and cholesterol synthesis, on PD. Such analysis would help to answer the question of whether statins exert a neuroprotective effect in PD, and if so, whether this effect depends on *HMGCR* genetic variability. The acquired knowledge could contribute to earlier diagnosis, better management of the disease, and to a possible individualisation of treatment.

## Figures and Tables

**Table 1:** Available data on the effect of statins on PD, the role of *HMGCR* variants in statin treatment, and unanswered questions in the field. PD—Parkinson’s disease, LDL-C—low-density lipoprotein cholesterol.

Statins in PD	Influence of *HMGCR* Variants on Statin Treatment	Questions Raised
statins exert protective function against PD [12,39]; significant protective effect is linked to atorvastatin [39]; progression of the motor symptoms in PD is slower in statin users [68]; statin users experience lower cognitive decline [67].	haplotype 7 (rs17244841, rs17238540, and rs3846662) is associated with reduced change in LDL-C level upon statin treatment [71,72]; conflicting results of *HMGCR* rs3846662 major allele (correlated with splicing defect) in terms of statin treatment responses [75,76,77]; *HMGCR* rs17238540 polymorphism is associated with lower reduction in total cholesterol level [78].	neuroprotive actions of statins may differ depending on the statin used [12,39]; ambiguous role of cholesterol in PD [13,24,25,26,29,31,33,34,35]; *HMGCR* variants may influence baseline LDL-C level [77], statin treatment [71,72,75,76,77,78] and possibly their neuroprotective effects.
